# A Clinical Analysis of Risk Factors for Interstitial Lung Disease in Patients with Idiopathic Inflammatory Myopathy

**DOI:** 10.1155/2013/648570

**Published:** 2013-09-09

**Authors:** Xiaomin Cen, Chuan Zuo, Min Yang, Geng Yin, Qibing Xie

**Affiliations:** Department of Rheumatology and Immunology, West China Hospital of Sichuan University, Chengdu, Sichuan 610041, China

## Abstract

Interstitial lung disease (ILD) is a common and severe complication of idiopathic inflammatory myopathies (IIM). The aim of our study was to identify risk factors for ILD by evaluating both clinical and biochemical features in IIM patients with or without ILD. From January 2008 to December 2011, medical records of 134 IIM patients in our rheumatology unit were reviewed. The patients were divided into ILD group (83 patients) and non-ILD group (51 patients). The clinical features and laboratory findings were compared. The univariable analyses indicated that arthritis/arthralgia (54.2% versus 17.6%, *P* < 0.05), Mechanic's hand (16.9% versus 2.0%, *P* < 0.05), Raynaud's phenomenon (36.1% versus 2.0%, *P* < 0.05), heliotrope rash (44.6% versus 19.6%, *P* < 0.05), fever (43.4% versus 21.6%, *P* < 0.05), elevated ESR (60.2% versus 35.3%, *P* < 0.05), elevated CRP (55.4% versus 31.4%, *P* < 0.05), or anti-Jo-1 antibody (20.5% versus 5.9%, *P* < 0.05) were risk factors for developing ILD in IIM. Multivariable unconditional logistic regression analysis that showed arthritis/arthralgia (OR 7.1, 95% CI 2.8–18.1), Raynaud's phenomenon (OR 29.1, 95% CI 3.6–233.7), and amyopathic dermatomyositis (ADM) (OR 20.2, 95% CI 2.4–171.2) were the independent risk factors for developing ILD in IIM.

## 1. Introduction

Idiopathic inflammatory myopathies (IIM) is a systemic autoimmune disease with unknown origin, characterized by proximal, symmetric muscle weakness, elevated serum creatine kinase (CK), characteristic electromyography findings, and lymphocytic infiltration in the muscle tissue [1, 2]. Polymyositis (PM) and dermatomyositis (DM) are the most common forms of IIM. In addition, amyopathic dermatomyositis (ADM) is a special type of DM [3]. In the case of DM, characteristic skin manifestations (heliotrope rash/Gottron papules) are also present [1]. PM and DM occur isolated or in connection with other connective tissue diseases (CTD) or malignancy [4].

 Interstitial lung disease (ILD) is a common and severe complication of IIM. Concurrency of ILD in IIM has been reported to be 23.1–65% and considered the major cause of death [5]. Since ILD is associated with unfavorable clinical outcome, it requires more aggressive medications as corticosteroids and immunosuppressive drugs. The reported frequency of ILD is more than 70% in Jo-1 positive patients [6]. Anti-Jo-1 antibody can be found in 10–40% of patients with polymyositis (PM), 2–10% in dermatomyositis (DM), and 3–8% in overlap myositis [7]. The presence of this autoantibody helps to identify a subgroup of patients characterized by ILD, Raynaud's phenomenon, arthritis, and “mechanic's hand,” referred to as “antisynthetase syndrome” [8, 9]. Anti-SS-A antibody can be found in 44–58% of patients with Jo-1 positive [10, 11]. Whether ILD in IIM is related to certain factors needs further research. The aim of this retrospective study was to investigate risk factors for ILD in patients with IIM.

## 2. Patients and Methods

134 patients with IIM from inpatient and outpatient department of our rheumatology unit between January 2008 and December 2011 were retrospectively reviewed. Diagnosis of PM (58 cases)/DM (58 cases)/ADM (18 cases) was established according to the criteria of ENMC workshop [3]. Patients who had other connective tissue diseases or malignancy concomitantly were excluded. Diagnosis of ILD was established based on the results of high-resolution computed tomography (HRCT).

 Clinical data was obtained from patients' medical records. All patients underwent detailed laboratory examinations and clinical assessment to exclude malignancy and other connective tissue disease. Disease duration was determined from date of diagnosis to the latest follow-up visit. The clinical features include age, sex, proximal muscle weakness, myosalgia, arthritis/arthralgia, mechanic's hand, Raynaud's phenomenon, Gottron's sign, heliotrop rash, and fever. All patients also underwent routine laboratory examinations at diagnosis: CK and erythrocyte sedimentation rate (ESR) were detected by enzyme rate method and Westergren method, respectively. Laser nephelometry was used to detect the presence of C-reactive protein (CRP) (Dialab GmBH, Austria). Antinuclear antibodies (ANA) were detected by indirect immunofluorescence method using Hep-2 cell as substrate. Antibodies directed against extractable nuclear antigen (ENA) complex SS-A and Jo-1 were measured by immunoblotting (Euroline-WB, Euroimmun, Lübeck, Germany). All patients underwent electromyography (EMG) examination. The presence of polyphasic, short, small motor unit potentials, fibrillation, positive sharp waves, and repetitive high frequency discharges was considered typical of IIM changes. After informed consent, all patients underwent muscle biopsy.

 Statistical analyses were performed with the SPSS version 19.0 software; *P* value was set at less than 0.05. The groups were analyzed with the following tests. In case of normal distribution the independent sample *t* test was used, and in nonnormal distribution Mann-Whitney test was adopted to compare the means. The chi-square test or Fisher's exact test was used to compare frequencies. However, caution is needed in interpreting statistical significance given the relative small number of patients. The unconditional multivariable logistic regression analysis was adopted to identify the risk factors.

## 3. Results

A total of 134 IIM patients were enrolled, including 83 (64.2%) with ILD (mean age 46.6 ± 12.4, range 16–82) and 51 (35.8%) without (mean age 40.4 ± 11.9, range 16–72). No significant differences were found between the two groups with regard to age, gender, and disease duration (Table 1). 

The constituent ratio of three subtypes of IIM with ILD were summarized in Table 2. The constituent ratio was significantly different according to the result of chi-square test (chi-square value = 10.6, *P* < 0.05). It was no significant difference between PM with ILD and DM with ILD (chi-square value = 1.3, *P* = 0.26). ADM presented a statistically more frequent association with ILD than PM (chi-square value = 6.8, *P* < 0.05) and DM (chi-square value = 10.6, *P* < 0.05). 

Clinical symptoms at presentation were summarized in Table 1. In both groups, no significant differences were found regarding myosalgia and Gottron's sign. The IIM with ILD patients had a statistically more frequent presence of arthritis/arthralgia, mechanic's hand, Raynaud's phenomenon, heliotrop rash, and fever (*P* < 0.05). However, this group had a statistically less frequent presence of proximal muscle weakness (*P* < 0.05) than IIM without ILD group. 

 Elevated ESR was detected in 50 (60.2%) patients with ILD versus 18 (35.3%) patients without ILD, elevated CRP was detected in 46 (55.4%) patients with ILD versus 16 (31.4%) patients without ILD, and elevated CK was detected in 62 (74.7%) patients with ILD versus 47 (92.2%) patients without ILD. These data showed that IIM patients with ILD had statistically more frequent presence of elevated ESR and CRP, while in the other group elevated CK was more common. 

 The presence of ANA was not significantly different between patients with and without ILD (68.7% versus 66.7%). Anti-SS-A antibody was found in 24 (28.9%) patients with ILD versus 11 (21.6%) patients without ILD. The difference between the two groups did not reach a statistical significance. The presence of anti-Jo-1 antibody was statistically more frequent in ILD group (*P* < 0.05). Anti-SS-A antibody was found in 11 (55.0%) patients with anti-Jo-1 antibody positive versus 24 (21.1%) patients with anti-Jo-1 antibody negative (*P* < 0.05).

 Multivariable unconditional logistic regression analysis indicated that arthritis/arthralgia, Raynaud's phenomenon, and ADM were the independent risk factors for developing ILD in IIM (Table 3). 

## 4. Discussion 

IIM-related ILD was originally described by Mills and Mathews in 1956 [12]. In this study, we found that the concurrency of ILD in patients with IIM was 64.2% (83/134), which was consistent with previous reports [5]. The aim of our study was to identify risk factors for ILD in patients with IIM by evaluating clinical and biochemical features in IIM patients with and without ILD. The acknowledgement of predictors for ILD in IIM patients appears crucial to prompt management at an early stage of the disease. Our results showed that age, gender, and disease duration were incorrelation with ILD, which was similar to the published studies [13, 14]. As known before, arthritis/arthralgia and positive anti-Jo-1 were important predictive factors for ILD in IIM [14–19] and were suggested to constitute a distinct subgroup of myositis, which was named antisynthetase syndrome [8, 9]. In this study, we confirmed not only this viewpoint but also demonstrated that Mechanic's hand, Raynaud's phenomenon, heliotrop rash, fever, elevated ESR, and CRP were associated with ILD. However, anti-Jo-1, Mechanic's hand, heliotrop rash, fever, elevated ESR, and CRP were not the independent risk factors for developing ILD in IIM, which may attribute to the nature of disease, the multicollinearity of these factors, or the small sample in our study.

 ADM is a special type of DM. In our study, prevalence of ILD was found 94.4% (17/18) in ADM patients. In PM and DM, however, the ratio was much lower to 62.1% and 51.7%, respectively. Cases with characteristic DM rash and little or no muscle involvement are regarded as ADM [15]. As the results shown in our study, ADM subtype closely correlated with ILD, which may lead to poor prognosis [15, 20–22]. Therefore, early diagnosis of ILD and aggressive approach in therapy is required in patients with ADM.

Anti-Jo-1 antibody is known as a marker for myositis [23, 24] and is found to be positive in 20%–30% of patients with IIM [25]. Previous literature showed that the prevalence of ILD approached 70%–90% among anti-Jo-1 antibody positive individuals [6, 26]. In our cohort, the prevalence of anti-Jo-1 antibody was 14.9%, and the prevalence of ILD was 85.0%, which was similar to previous investigations. In addition, the association of anti-Jo-1 with anti-SS-A antibodies has been reported in the literature, suggesting the coexistence of positive anti-Jo-1 and anti-SS-A could serve as a good predictor to identify candidate patients for severe progressive ILD [7, 10, 27]. The similar conclusion was made in our study that the presence of anti-SS-A antibody was statistically more frequent in patients with anti-Jo-1 antibody positive. But anti-SS-A antibody was incorrelation with ILD by comparing the frequency of anti-SS-A antibody in patients with or without ILD. Therefore, although anti-SS-A antibody was associated with anti-Jo-1 antibody, it could not be considered as an independent predictive factor for developing ILD in IIM.

 In conclusion, our findings suggested that ILD was prevalent in patients with IIM, especially in patients with ADM. Patients who presented with arthritis/arthralgia and Raynaud's phenomenon tended to have a higher frequency of IIM-associated ILD. Furthermore, anti-SS-A antibody was associated with anti-Jo-1 antibody, but it was not an independent predictive factor for ILD in patients with IIM.

## Figures and Tables

**Table 1:** Univariable analysis of risk factors for ILD in patients with IIM.

Items	With ILD (*n* = 83)	Without ILD (*n* = 51)	*P* value
Mean age at diagnosis (years)	46.6 ± 12.4	40.4 ± 11.9	n.s.
Male : female	24 : 59	13 : 38	n.s.
Disease duration (months)	19.3 ± 7.6	22.5 ± 8.0	n.s.
Proximal muscle weakness	59 (71.1%)	45 (88.2%)	<0.05
Myosalgia	39 (47.0%)	25 (49.0%)	n.s.
Arthritis/arthralgia	45 (54.2%)	9 (17.6%)	<0.05
Mechanic's hand	14 (16.9%)	1 (2.0%)	<0.05
Raynaud's phenomenon	30 (36.1%)	1 (2.0%)	<0.05
Gottron's sign	33 (39.8%)	16 (31.4%)	n.s.
Heliotrope rash	37 (44.6%)	10 (19.6%)	<0.05
Fever	36 (43.4%)	11 (21.6%)	<0.05
Elevated ESR	50 (60.2%)	18 (35.3%)	<0.05
Elevated CRP	46 (55.4%)	16 (31.4%)	<0.05
Elevated CK	62 (74.7%)	47 (92.2%)	<0.05
ANA (+)	57 (68.7%)	34 (66.7%)	n.s.
Anti-SS-A antibody (+)	24 (28.9%)	11 (21.6%)	n.s.
Anti-Jo-1 antibody (+)	17 (20.5%)	3 (5.9%)	<0.05

IIM: idiopathic inflammatory myopathies; ILD: interstitial lung disease; ESR: erythrocyte sedimentation rate; CRP: C-reactive protein; CK: creatine kinase, ANA: antinuclear antibody; n.s.: not significant.

**Table 2:** The constituent ratio of three subtypes of IIM with ILD.

Subtypes	With ILD	Without ILD	Chi-square value	*P* value
PM	36 (62.1%)	22 (37.9%)	1.3*	0.26
DM	30 (51.7%)	28 (48.3%)	10.6**	<0.05
ADM	17 (94.4%)	1 (5.6%)	6.8***	<0.05

*PM versus DM, **DM versus ADM, and ***ADM versus PM

IIM: idiopathic inflammatory myopathies; ILD: interstitial lung disease; PM: polymyositis; DM: dermatomyositis; ADM: amyopathic dermatomyositis.

**Table 3:** Multivariable unconditional logistic regression analysis on risk factors associated with ILD in patients with IIM.

Variables	*B*	Wald	*P* value	OR	95.0% CI for OR
Arthritis/arthralgia	1.96	16.90	0.000	7.1	2.8–18.1
Raynaud's phenomenon	3.37	10.04	0.002	29.1	3.6–233.7
Amyopathic dermatomyositis	3.01	7.60	0.006	20.2	2.4–171.2

IIM: idiopathic inflammatory myopathies, ILD: interstitial lung disease, *B*: regression coefficient, OR: odds ratio, CI: credibility interval.
